# Barth syndrome

**DOI:** 10.1002/ajmg.c.31372

**Published:** 2013-07-10

**Authors:** JOHN L JEFFERIES

**Affiliations:** Associate Professor of Pediatric Cardiology and Adult Cardiovascular Diseases in the Heart Institute at the Cincinnati Children’s Hospital Medical Center. His clinical and research interests center on advanced therapies for advanced heart failure as well as novel drug and device therapies for myocardial dysfunction.

**Keywords:** Barth syndrome, genetics, heart failure, cardiomyopathy

## Abstract

Barth syndrome (BTHS) is an X-linked recessive disorder that is typically characterized by cardiomyopathy (CMP), skeletal myopathy, growth retardation, neutropenia, and increased urinary levels of 3-methylglutaconic acid (3-MGCA). There may be a wide variability of phenotypes amongst BTHS patients with some exhibiting some or all of these findings. BTHS was first described as a disease of the mitochondria resulting in neutropenia as well as skeletal and cardiac myopathies. Over the past few years, a greater understanding of BTHS has developed related to the underlying genetic mechanisms responsible for the disease. Mutations in the *TAZ* gene on chromosome Xq28, also known as G4.5, are responsible for the BTHS phenotype resulting in a loss-of-function in the protein product tafazzin. Clinical management of BTHS has also seen improvement. Patients with neutropenia are susceptible to life-threatening bacterial infections with sepsis being a significant concern for possible morbidity and mortality. Increasingly, BTHS patients are suffering from heart failure secondary to their CMP. Left ventricular noncompaction (LVNC) and dilated CMP are the most common cardiac phenotypes reported and can lead to symptoms of heart failure as well as ventricular arrhythmias. Expanded treatment options for end-stage myocardial dysfunction now offer an opportunity to change the natural history for these patients. Herein, we will provide a current review of the genetic and molecular basis of BTHS, the clinical features and management of BTHS, and potential future directions for therapeutic strategies. © 2013 Wiley Periodicals, Inc.

## INTRODUCTION

Barth syndrome (BTHS; OMIM #302060) was first described in 1983 as an X-linked disease of neutropenia, skeletal myopathy, and cardiomyopathy (CMP) in a large Dutch pedigree with high infant mortality [Barth et al., 1983]. However, the initial description may have occurred in 1979 by Neustein and coworkers reporting on an infant with CMP and heart failure who was found to have abnormal mitochondria in the heart, liver, kidney, and skeletal muscle [Neustein et al., 1979]. These patients were noted to have abnormal appearing mitochondria and the patients died early in life from septicemia or heart failure. Subsequent genetic studies have identified that BTHS is secondary to loss-of-function mutations in the *TAZ* gene (G4.5) located on chromosome Xq28 which encodes for tafazzin. Tafazzin is a phospholipid transacylase which is located in the inner leaflet of the mitochondrial membrane and plays an important role in the remodeling of cardiolipin (CL). CL is known to be critical in maintaining mitochondrial structure as well as being involved in mitochondrial apoptosis [Koshkin and Greenberg, 2002; Gonzalvez and Gottlieb, 2007].

**Tafazzin is a phospholipid transacylase which is located in the inner leaflet of the mitochondrial membrane and plays an important role in the remodeling of cardiolipin (CL). CL is known to be critical in maintaining mitochondrial structure as well as being involved in mitochondrial apoptosis.**

BTHS is a rare disorder with less than 500 patients in the BTHS Registry from around the world. There are no known ethnic or racial predilections. It is estimated that there are ∼10 new cases of BTHS diagnosed in the US each year. Although the actual prevalence is unknown, the Barth Syndrome Foundation reports an estimate of 1/300,000–400,000 live births. There are increasing data to suggest that BTHS may be underdiagnosed with a possible prevalence as high as 1/140,000 live births [Cantlay et al., 1999].

Although rare, the clinical implications of BTHS can be devastating. Heightened awareness of this disease is needed globally as timely diagnosis may afford patients access to appropriate therapies and improved outcome.

## GENETICS

The tafazzin (*TAZ*) gene, also known as G4.5, located in the long q arm region of chromosome Xq28, is responsible for BTHS [Bione et al., 1996; Johnston et al., 1997]. The TAZ gene is highly evolutionarily conserved [D’Adamo et al., 1997]. It is comprised of 11 exons, the first two of which are noncoding, and spans ∼11 kb [Bolhuis et al., 1991]. There are two alternative translation start sites which results in alternative splice forms. More than 160 mutations have been identified and involve every exon of the TAZ gene [Gonzalez, 2005]. The majority of reported mutations are small insertions or deletions as well as missense mutations. Large deletions and truncation mutations have also been reported. A complete list of reported mutations is maintained and updated by the Barth Syndrome Foundation (http://www.barth-sydrome.org). Less than 20% of boys carry de novo mutations not identified in maternal somatic DNA but gonadal mosacisim has been reported [Chang et al., 2010]. There are no reported genotype/phenotype correlations in BTHS and it is increasingly recognized that there may be significant intrafamilial phenotypic variability [Ronvelia et al., 2012]. The use of next generation sequencing (NGS) has recently been reported as a possible diagnostic strategy in BTHS. Man et al. employed NGS to identify a hemizygous variant in the TAZ gene in two male siblings with infantile dilated cardiomyopathy (DCM) [Man et al., 2013].

## PATHOPHYSIOLOGY

**Figure 1:**
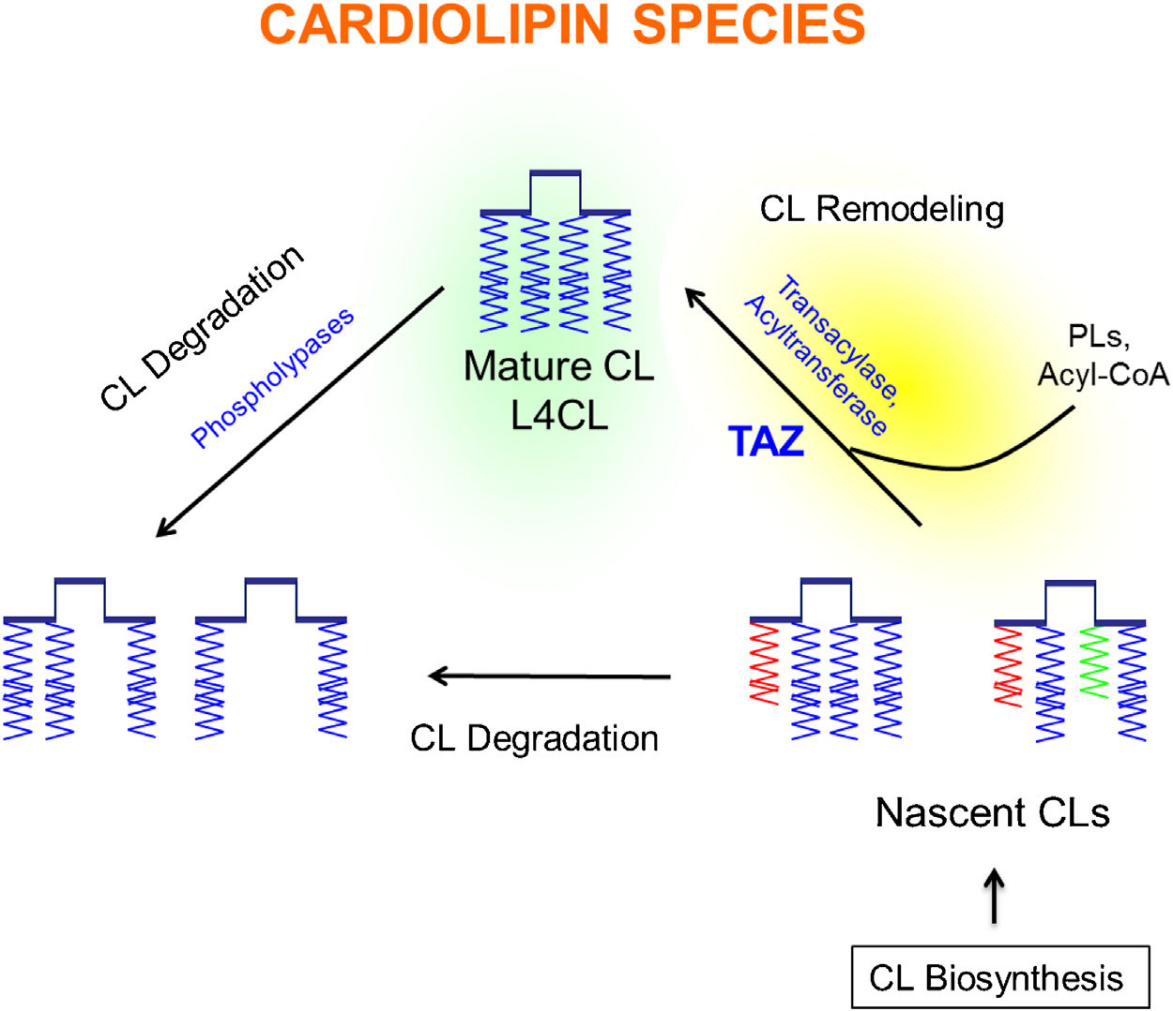
During its biosynthesis, cardiolipins are made as immature forms with different non-symetric acyl groups. During remodeling, these different acyl groups are replaced with lynoleic acids and 80% of CL in cardiac and muscle cells is symmetric L4CL. In contrast, brain has very large diversity of CL molecular forms. Mutations in the *TAZ* gene cause BTHS. BTHS patients have deficiency of mature L4CLs and accumulation of immature CLs and lysocardiolipins, or CLs missing one or two acyl groups. (Image generously supplied by Zaza Khuchua, Division of Molecular and Cardiovascular Biology, Cincinnati Children’s Research Foundation, Cincinnati, OH.)

*TAZ* encodes a phospholipid acyltransferase that is involved in the remodeling of CL which is located on the inner mitochondrial membrane [Neuwald, 1997; Vreken et al., 2000]. CL has alternative fatty acyl chain configurations that are tissue specific. Tissues that are highly oxidative such as cardiac and skeletal muscle normally have a CL with four linoleoyl species (tetralinoleoyl cardiolipin or L4-CL). *TAZ* mutations result in reduced formation of L4-CL and an increase in intermediate species of CL that carry three linoleoyl acyl groups which are referred to as monolysocardiolipins (MLCL). Tafazzin catalyzes the remodeling of immature CL after initial synthesis to mature CL which is predominantly L4-CL. In BTHS with abnormalities in tafazzin, less mature CL is produced resulting in the ratio of MLCL:L4-CL being increased [Schlame et al., 2002; Valianpour et al., 2005] (Fig. 1).

CL has multiple roles as mentioned above, including maintenance of mitochondrial structure and involvement in mitochondrial apoptosis but also is needed to optimize cellular energy production [Klingenberg, 2009]. CL is thought to be critical in the formation of mitochondrial cristae as well as mediating assembly and stabilizing electron transport chain complexes [Acehan et al., 2007; Kiebish et al., 2008].

## CLINICAL FEATURES

Patients may have some or all of the reported associated clinical features of BTHS. The most widely reported of these are CMP, skeletal myopathy, growth delay, neutropenia, and increased urinary excretion of 3-methylglutaconic acid (3-MGCA). In an attempt to better characterize the clinical features of BTHS, the BTHS Registry in conjunction with the Barth Syndrome Foundation (BSF) was created in 2006. Some of the data collected from the Registry are described below which includes self-reported or medical chart abstraction data on 73 subjects. Based on these and other data, Roberts et al. [2012] recognized that the phenotypic presentation may be variable in an individual patient and across the BTHS population over time. Of the 73 patients in the Registry, 62 reported a total of 250 hospitalizations. The majority of these were related to infection and cardiac complications (Table1).

***In an attempt to better characterize the clinical features of BTHS, the BTHS Registry in conjunction with the Barth Syndrome Foundation (BSF) was created in 2006. Some of the data collected from the Registry are described below which includes self-reported or medical chart abstraction data on 73 subjects.***

**Table I:** Clinical Findings Described in Barth Syndrome (BTHS)

Cardiovascular	Dilated cardiomyopathy (DCM)
	Left ventricular noncompaction (LVNC)
	Hypertrophic cardiomyopathy (HCM)
	Mixed cardiomyopathy phenotype
	Undulating form of cardiomyopathy
	Endocardial fibroelastosis (EFE)
	Ventricular arrhythmias/sudden cardiac death (SCD)
	Cerebrovascular accidents (thromboembolic events)
Metabolic and endocrine	3-MGCA aciduria
	Delayed bone age
	Growth delay
	Delayed onset of puberty
	Osteopenia
	Hypocholesterolemia
Dysmorphisms	Full cheeks
	Deep set eyes
	Prominent ears
Hematologic	Neutropenia
Infectious	Recurrent oral ulcers
	Perianal dermatitis
Skeletal muscle	Proximal myopathy
	Easy fatigability
	Exercise intolerance
Developmental	Learning disabilities
	Oromotor feeding abnormalities
	Attention deficit disorder
	Delayed motor milestones
Fetal	Miscarriage/stillbirth
	Cardiomyopathy
	Heart failure/hydrops

### Neutropenia

Neutropenia is very common in BTHS and may be persistent or intermittent. However, there is a wide range of clinical findings with some patients having extremely low levels of neutrophils and others having only mild involvement to normal levels of circulating neutrophils. Neutropenia was reported in 69.1% of 73 patients from the BTHS Registry [Roberts et al., 2012]. Only 25% of the cohort reported no prior diagnosis of neutropenia. Neutropenia may be the initial finding in an undiagnosed BTHS patient and may be seen prior to birth [Barth et al., 1983]. Myelocyte arrest is seen on bone marrow aspiration with neutrophils showing membrane-bound vacuoles [Barth et al., 1983]. The exact mechanism behind neutropenia in BTHS remains unclear. Although it has been suggested that the mitochondrial defects are responsible for neutropenia leading to apoptotic mechanisms and increased macrophage clearance, no definitive results have been published [van Raam and Kuijpers, 2009; Finsterer and Frank, 2013]. Importantly, neutrophil killing activity and directed motility is normal in BTHS although total neutrophil numbers may be depressed [Kuijpers et al., 2004].

***Neutropenia may be the initial finding in an undiagnosed BTHS patient and may be seen prior to birth.***

***Myelocyte arrest is seen on bone marrow aspiration with neutrophils showing membrane-bound vacuoles.***

***The exact mechanism behind neutropenia in BTHS remains unclear.***

The clinical implications of neutropenia can be life-threatening secondary to possible bacterial infection and resultant sepsis. Clinical vigilance must be high as neutropenia may develop in someone who otherwise has had low normal to mild decrease in circulating levels. Ongoing surveillance is critical for a good outcome as levels may fluctuate dramatically in a single patient. Neutropenia is responsible for other morbidity leading to respiratory infections, dermatologic infections, and mouth ulcers.

### Growth Delay

Growth delay is a well-described clinical feature of BTHS. The BTHS Registry evaluated 764 growth points on 60 patients and superimposed these points on the CDC growth curves for normal age appropriate males [Roberts et al., 2012]. Between ages 6 and 36 months, the 50th centile for length in BTHS boys overlaps the 3rd centile on the normative curve. After age 12–14, there is an increase in growth velocity for height compared both to the normative population as well as to their previous velocity. These data are consistent with those previously reported by Spencer et al. [2006] that ∼60% of BTHS boys less than 18 years of age were below the 5th centile for height. For clinical management, normalized BTHS growth charts have been generated based on data from the BTHS Registry.

### Cardiomyopathy and Cardiac Arrhythmias

**Figure 2:**
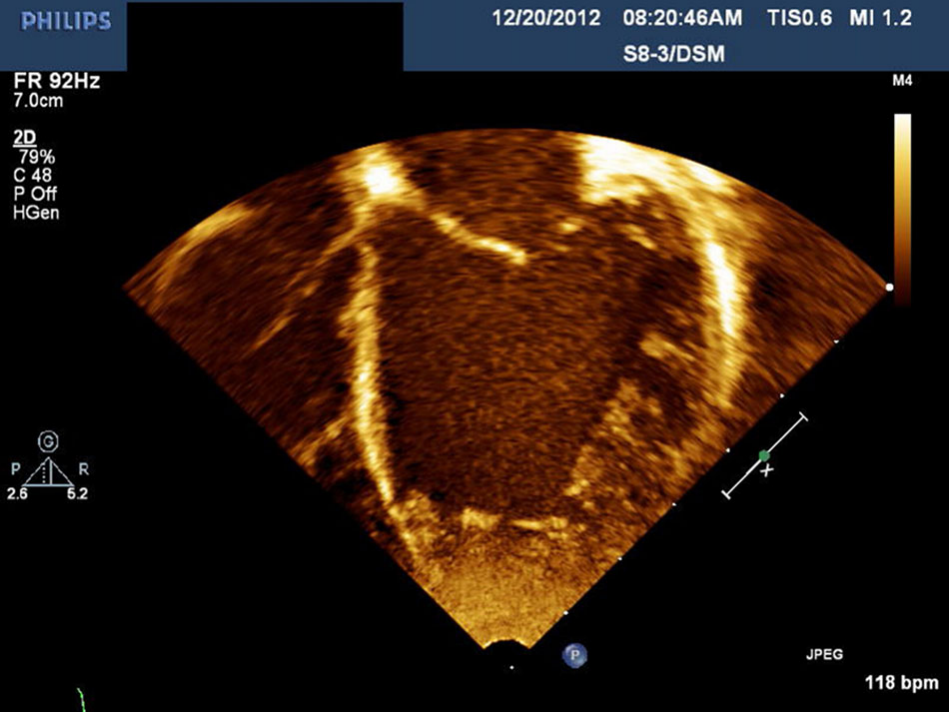
Echocardiogram (apical 4-chamber view) of patient with BTHS depicting LVNC with associated DCM phenotype. Note the deep LV trabeculations and dilated left ventricular chamber.

Cardiac disease is common in BTHS. BTHS Registry data describe 69 of 73 subjects reporting a history of CMP [Roberts et al., 2012]. Of these, ∼70% reported being diagnosed within the first year of life. The clinical phenotype of TAZ mutations may be quite heterogeneous. DCM is a common cardiac phenotype, characterized by decreased left ventricular (LV) systolic function, increased LV mass, and an increased LV end-diastolic dimension [Jefferies and Towbin, 2010]. Left ventricular noncompaction (LVNC) is also commonly seen either alone or in conjunction with other CMP phenotypes and is characterized by trabeculations in LV with associated wall motion abnormalities [Towbin, 2010] (Fig. 2). The incidence of LVNC or LV hypertrabeculation (LVHT) in BTHS is not known. However, it is likely that the number of diagnoses will increase given the advancements in imaging and increasing use of cardiac MRI as well as increased awareness of LVNC/LVHT. Endocardial fibroelastosis (EFE) may be seen, although less commonly [Ades et al., 1993]. Hypertrophic cardiomyopathy (HCM) has been reported in association with BTHS [Bleyl et al., 1997]. Patients diagnosed previously with the apical form of HCM should be carefully assessed as this may be confused clinically with LVNC and would potentially alter management. A mixed hypertrophic-dilated cardiac phenotype characterized by thickening of the LV walls with an increase in LV mass and end-diastolic dimension and depressed systolic function has also been reported [Towbin and Bowles, 2002]. Transition between distinct phenotypes has also been described in the setting of LVNC which has been termed an “undulating phenotype” [Pignatelli et al., 2003]. No current mechanism has been proposed that explains the various CMP phenotypes seen in BTHS. However, evidence of varying phenotypic cardiac disease is well documented in families with recognized sarcomeric mutations suggesting shared molecular etiology of different forms of CMP [Klaassen et al., 2008]. Unfortunately, the diagnosis of BTHS is often not considered in patients presenting with CMP. Consideration of BTHS should be given to all males presenting with CMP, especially those presenting at a young age. Nugent et al. [2003] reported 4.8% of boys diagnosed with CMP in Australia between the years of 1987–1996 had BTHS underscoring the importance of a complete evaluation of possible etiologies of CMP in young males.

***DCM is a common cardiac phenotype, characterized by decreased left ventricular (LV) systolic function, increased LV mass, and an increased LV end-diastolic dimension.***

***Left ventricular noncompaction (LVNC) is also commonly seen either alone or in conjunction with other CMP phenotypes and is characterized by trabeculations in LV with associated wall motion abnormalities.***

In addition, there is an increased risk of arrhythmias in BTHS, some of which may be life-threatening. These may be a result of abnormal mitochondrial function but also may be a function of the associated cardiac phenotype as ventricular arrhythmias are well reported in DCM, LVNC, and HCM [Jefferies and Towbin, 2010; Gersh et al., 2011; Brescia et al., 2013].

### Female Carriers

Previously, it has been thought that female carriers of would not exhibit clinical features of BTHS due to the presence of a normally functioning wild-type gene in the setting of an X-linked disease. However, clinical findings of BTHS with genetic confirmation of deletions of exons 1 through 5 in the *TAZ* gene were reported in a female infant [Cosson et al., 2012]. Respiratory chain analysis performed on skin fibroblasts revealed decreased activity of complexes I, III, and IV. Subsequent cytogenetic analysis revealed mosaicism for monosomy X and a ring X chromosome with a large deletion of which included the Xq28 region. The patient had LVNC with an associated DCM phenotype by echocardiography as well as neutropenia. She had evidence of heart failure at age 1 month and died at age 3 from sepsis.

## DIAGNOSIS

Historically, BTHS has been diagnosed based on clinical presentation relying heavily on identifying boys with neutropenia, CMP, skeletal myopathy, and elevated urinary levels of 3-MGCA. However, based on the highly variable clinical presentations in BTHS, utilizing this approach make reliable diagnosis problematic. Armed with a greater understanding of CL species in BTHS, Houtkooper et al. [2009] developed a diagnostic assay which measures the MLCL:L4-CL ratio. This test can be performed on a variety of tissues including stored bloodspot cards with a reported sensitivity and specificity of 100%. However, access to this testing remains quite limited and although clinically promising, is not used broadly. Alternatively, genetic testing on *TAZ* can be performed at commercial laboratories both nationally and internationally and facilitate a diagnosis. Most available commercial panels for testing of patients with either LVNC or DCM typically include *TAZ* screening.

## MANAGEMENT

Previously, BTHS was often considered a lethal infantile and early childhood disease [Christodoulou et al., 1994]. However, improvements in management of associated neutropenia/infectious risks, skeletal myopathy, and cardiac disease have resulted in better survival. Although mortality is highest in the first 4 years of life, patients are reported living into their late 40s [Barth et al., 2004] and a report of a single patient surviving to age 51 [Ronvelia et al., 2012]. This is in large part secondary to treatment of low circulating neutrophil counts and avoidance of infection, systolic dysfunction, and cardiac arrhythmias, as described below. Interestingly, a clear change in survival has recently been reported based on birth year with patients born after 2000 having a 70% survival rate compared with a 20% survival rate in those born before 2000 [Rigaud et al., 2013]. This suggests improved management strategies are having a measurable effect on outcome.

***Interestingly, a clear change in survival has recently been reported based on birth year with patients born after 2000 having a 70% survival rate compared with a 20% survival rate in those born before 2000***

***This suggests improved management strategies are having a measurable effect on outcome.***

### Management of Neutropenia

Granulocyte colony stimulating factor (G-CSF) has been widely used in BTHS with reasonable success. G-CSF is typically used in times of neutropenia concomitant with appropriate prophylactic antibiotics if clinically indicated. Treatment is often used to raise counts to near normal and may not be continued indefinitely given the wide variability in counts that may be seen. G-CSF may be given subcutaneously either twice weekly or every other day based on severity. BTHS Registry data report nearly 50% of the cohort had been on G-CSF at some point in their history with 27.5% being actively treated [Roberts et al., 2012].

### Management of Growth Delay

Although decreased levels of growth hormone (GH) have been reported in BTHS patients less than 15 years, levels typically are higher than normal controls in the late teens and early twenties. As a result, GH supplementation has not been routinely used unless documented central GH deficiency has been established. Arginine depletion has been implicated in possibly contributing to low growth rates in BTHS. Low levels of arginine have been documented in the serum of BTHS patients and may result in limitations in protein synthesis [Coman et al., 2008; Rigaud et al., 2013]. This has resulted in an increased use of arginine supplementation as a possible treatment to improve growth rates.

### Management of LV Systolic Dysfunction and Heart Failure

Treatment of associated myocardial dysfunction is paramount to alleviating symptoms as well as prolonging life. Medical and surgical options for DCM in BTHS have increased over the past few years and are largely driven by treatments used for adults with HF as well as children with DCM from other causes. Medical therapies must be directed at the particular cardiac phenotype being managed. Typical therapy for DCM would consist of angiotensin converting enzyme inhibitors (ACEi) or alternatively angiotensin receptor blockers (ARB) in combination with approved beta blockers [Jessup and Brozena, 2007]. Advanced medical therapies such as potassium sparing diuretics and digoxin may be considered in individual cases. For those patients with worsening HF, more aggressive therapies may be needed in the form of intravenous agents such as vasodilators or inotropes. The drugs may help to compensate HF and allow for transition back to chronic oral therapies and are to be used sparingly. However, there are some patients that require chronic inotropic support. In these scenarios, consideration may be given to mechanical circulatory support (MCS) and possible cardiac transplantation. Given the broad age spectrum currently of BTHS patients, there are numerous MCS strategies that are available which provide temporary or durable support for patients that have decompensated HF. For adolescents and young adults, larger devices are available that can effectively bridge patients to transplant [Jefferies and Morales, 2012]. Recent advances in technology have led to the development of MCS strategies for infants and children in the form of the Berlin EXCOR device which has received FDA approval for widespread use [Fraser et al., 2012]. This device can be used for an extended period of time even in the setting of neutropenia [Dedieu et al., 2013]. We recently used this device to support both the right ventricle and left ventricle in a patient with BTHS and severe biventricular dysfunction as a bridge to cardiac transplant [Hanke et al., 2012]. For those patients with refractory HF or those on MCS, cardiac transplant is typically considered. Although there are clinical concerns of neutropenia in the setting of required immunosuppression, we and others have reported success in selected cases [Mangat et al., 2007]. Nine patients in the BTHS Registry were successfully transplanted with a mean age at time of transplant of 3.8 ± 5.3 years (range: 3 months to 16 years) [Roberts et al., 2012]. Routine noninvasive imaging in the form of echocardiography or magnetic resonance imaging (MRI) should be part of routine management to assess for myocardial function as well as chamber size and wall thickness to help delineate appropriate therapy. And, with increasing options for the management of advanced HF, the need for cardiac transplantation may be obviated in the future [Rigaud et al., 2013].

### Management of Cardiac Arrhythmias

Although much attention is given to associated CMP with BTHS, clinically significant arrhythmias can be a major cause of mortality. The risk of ventricular arrhythmias is well known and may be precipitated by associated metabolic acidosis or concomitant LV systolic dysfunction [Yen et al., 2008]. Ventricular arrhythmias in the form of ventricular tachycardia (VT) or ventricular fibrillation (VF) may result in sudden cardiac death (SCD). The use of implantable cardioverter defibrillators (ICDs) has been documented in BTHS although limited data exist regarding effectiveness of this therapy [Spencer et al., 2005]. Life threatening arrhythmias may occur at any age but appears to be more common in older children. SCD has been reported to be the presenting symptom of BTHS and should be considered in the differential diagnosis of patients unknown to have BTHS presenting with a personal or family history of ventricular tachyarrhythmias, syncope, aborted sudden death, or SCD. Prolongation of the corrected QT interval has also been reported in BTHS which may be reflective of abnormal calcium handling or secondary to associated cardiac phenotypes such as HCM or DCM [Spencer et al., 2006; Jefferies and Towbin, 2010; Acehan et al., 2011]. Given that clinically significant arrhythmias may manifest at any time, ongoing clinical surveillance in the form of routine electrocardiograms and Holter monitoring should be part of clinical management.

## FUTURE DIRECTIONS

Ongoing efforts to better understand BTHS in humans have been historically limited secondary to the inability to develop a mammalian model. However, in 2010 an inducible *TAZ* knockdown model was developed [Acehan et al., 2011]. Many of the clinical features seen in humans such as CMP and skeletal myopathy as well as reductions in L4-CL and increases in the MLCL:L4-CL ratio. This mouse model offers some potential opportunities to better understand the pathophysiology of BTHS as well as rapidly study therapeutic interventions. One such intervention may be the use of induced pluripotent stem cells (iPS) in this population.

Advancements in the treatment of cardiac dysfunction and heart failure continue to be reported. Medical and technological breakthroughs that are developed and refined in adult ischemic and nonischemic CMP populations will be available to treat BTHS patients with cardiac disease and potentially prolong life without the need for cardiac transplantation.

## CONCLUSIONS

BTHS, although a rare disease, has seen evidence of impactful research and clinical intervention over the past few years. Diagnostic strategies are improving and therapeutic options, especially for cardiac disease, are expanding. Perhaps most importantly, the wide variability in BTHS phenotypes is being recognized and thoughtful, longitudinal care plans are being developed to avoid potentially catastrophic scenarios such as sepsis, decompensated heart failure, and SCD.

Cardiovascular disease in BTHS may come in the form of myocardial dysfunction or life-threatening arrhythmias. Management of systolic dysfunction may be effective with oral therapies but in some patients, advanced therapies may be necessary in the form of intravenous medications, MCS, and/or cardiac transplantation. The use of advanced device therapies such as ICDs has been reported and may be effective in managing patients with significant risk of life-threatening arrhythmias. Importantly, ongoing cardiac surveillance must be performed regularly as development of these sources of morbidity and mortality may develop at any time.

On a broader note, mitochondria have become a therapeutic target in the treatment of advanced heart failure in those patients without BTHS [Bayeva et al., 2013]. Heart failure, regardless of etiology, results in significant mortality with one in every five patients dying within 1 year of diagnosis [Lloyd-Jones et al., 2010]. Lessons learned from the management of BTHS may offer insight into treatment strategies for the millions living with heart failure world-wide.

Active investigation continues to occur in the basic, translational, and clinical research domains directed at better diagnostic and therapeutic strategies for the care of BTHS patients. Efforts must be increased to heighten awareness of this disease to facilitate earlier diagnosis and intervention and improve outcome.
